# Regional and seasonal variations in household and personal exposures to air pollution in one urban and two rural Chinese communities: A pilot study to collect time-resolved data using static and wearable devices

**DOI:** 10.1016/j.envint.2020.106217

**Published:** 2021-01

**Authors:** Ka Hung Chan, Xi Xia, Kin-fai Ho, Yu Guo, Om P Kurmi, Huaidong Du, Derrick A Bennett, Zheng Bian, Haidong Kan, John McDonnell, Dan Schmidt, Rene Kerosi, Liming Li, Kin Bong Hubert Lam, Zhengming Chen, on behalf of the CKB-Air Collaborative Group

**Affiliations:** a Clinical Trial Service Unit and Epidemiological Studies Unit, Nuffield Department of Population Health, University of Oxford, UK; b Jockey Club School of Public Health and Primary Care, The Chinese University of Hong Kong, Hong Kong Special Administrative Region; c Chinese Academy of Medical Sciences, China; d Faculty Research Centre for Intelligent Healthcare, Faculty of Health and Life Sciences, Coventry University, UK; e MRC Population Health Research Unit, Nuffield Department of Population Health, University of Oxford, UK; f School of Public Health, Fudan University, China; g Department of Epidemiology and Biostatistics, Peking University, China

**Keywords:** Exposure assessment, Household air pollution, Ambient air pollution, Solid fuels, Time-activity

## Abstract

**Background:**

Previous studies of the health impact of ambient and household air pollution (AAP/HAP) have chiefly relied on self-reported and/or address-based exposure modelling data. We assessed the feasibility of collecting and integrating detailed personal exposure data in different settings and seasons.

**Methods/design:**

We recruited 477 participants (mean age 58 years, 72% women) from three (two rural [Gansu/Henan] and one urban [Suzhou]) study areas in the China Kadoorie Biobank, based on their previously reported fuel use patterns. A time-resolved monitor (PATS+CO) was used to measure continuously for 120-hour the concentration of fine particulate matter (PM_2.5_) at personal and household (kitchen and living room) levels in warm (May-September 2017) and cool (November 2017–January 2018) seasons, along with questionnaires on participants’ characteristics (e.g. socio-demographic, and fuel use) and time-activity (48-hour). Parallel local ambient monitoring of particulate matter (PM_1_, PM_2.5_ and PM_10_) and gaseous pollutants (CO, ozone, nitrogen oxides) was conducted using regularly-calibrated devices. The air pollution exposure data were compared by study sites and seasons.

**Findings:**

Overall 76% reported cooking at least weekly (regular-cooks), and 48% (urban 1%, rural 65%) used solid fuels (wood/coal) for cooking. Winter heating was more common in rural sites than in urban site (74–91% vs 17% daily), and mainly involved solid fuels. Mixed use of clean and solid fuels was common for cooking in rural areas (38%) but not for heating (0%). Overall, the measured mean PM_2.5_ levels were 2–3 fold higher in the cool than warm season, and in rural (e.g. kitchen: Gansu_warm_season_ = 142.3 µg/m^3^; Gansu_cool_season_ = 508.1 µg/m^3^; Henan_warm_season_ = 77.5 µg/m^3^; Henan_cool_season_ = 222.3 µg/m^3^) than urban sites (Suzhou_warm_season_ = 41.6 µg/m^3^; Suzhou_cool_season_ = 81.6 µg/m^3^). The levels recorded tended to be the highest in kitchens, followed by personal, living room and outdoor. Time-resolved data show prominent peaks consistently recorded in the kitchen at typical cooking times, and sustained elevated PM_2.5_ levels (> 100 µg/m^3^) were observed in rural areas where use of solid fuels for heating was common.

**Discussion:**

Personal air pollution exposure can be readily assessed using a low-cost time-resolved monitor in different settings, which, in combination with other personal and health outcome data, will enable reliable assessment of the long-term health effects of HAP/AAP exposures in general populations.

## Highlights

• We collected detailed fuel use, time-activity, and air pollution data from 477 Chinese adults.
• Mix of solid and clean fuels was common for cooking or heating in rural areas.
• Real-time PM_2.5_ data at personal, household, and ambient environments are presented.
• PM_2.5_ levels in rural areas with solid fuel use were 2–3 times higher than in urban areas.
• Personal, household and ambient PM_2.5_ levels were 2–3 times higher in the cool season.

## Graphical abstract

Averaged 24-hour variation of fine particulate matter (PM_2.5_) concentrations (µg/m^3^) recorded in the personal, kitchen, living room, and ambient monitors across the three study sites in the warm and cool season.

**Figure:**
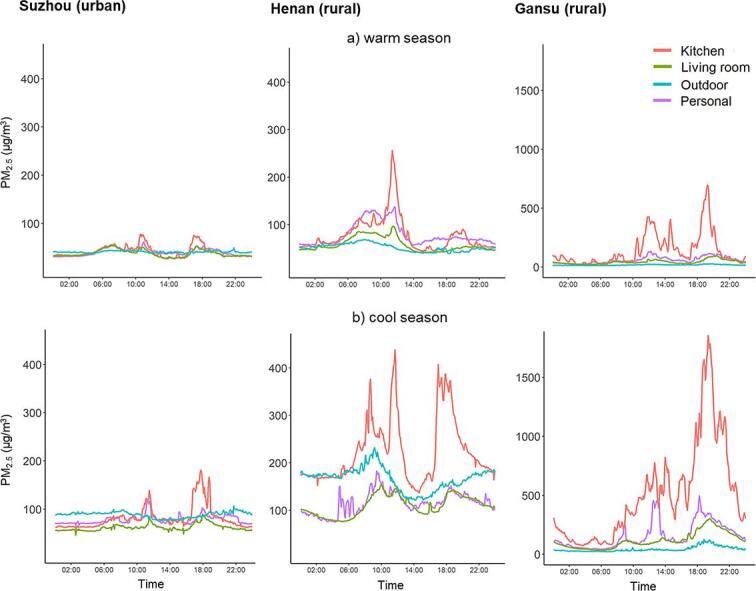

## 1 Background and rationale

The recent rapid urbanisation and industrialisation in many low- and middle-income countries (LMICs) has resulted in a notable rise in ambient air pollution (AAP) and a considerable decline in the proportion of exposure to household air pollution (HAP) from solid fuel use, yet the number exposed to HAP remained substantial at over 3.6 billion in 2018. (Health Effect Institute, 2019) Therefore, many LMICs including China (with 450 million solid fuel users in 2017) face a “double burden” of HAP and AAP. (Health Effect Institute, 2019, Zhao et al., 2018) Although the epidemiological evidence on the health impact of air pollution remains to be improved, it has been estimated that AAP and HAP together account for over 7 million premature deaths annually worldwide. (Landrigan et al., 2017).

Both short- and long-term exposure to AAP have been associated with excess risks of cardio-respiratory disease. (Landrigan et al., 2017) However, although LMICs now experience worse AAP, the vast majority of existing evidence on AAP were from high-income countries (HICs) with relatively low exposure levels. (Newell et al., 2017) This has resulted in substantial uncertainties about the exposure–response relationships between AAP and cardio-respiratory diseases, especially at high exposure levels. (Burnett et al., 2018) Moreover, most previous epidemiological studies relied on modelled proxy measures of AAP levels around individuals’ residential address (or communities), rarely with validation from actual personal or indoor exposure measurements even though people spend most of their time indoors. (Duan et al., 2015, Klepeis et al., 2001, Brauer et al., 2016) Previous epidemiological studies on HAP mostly focused on respiratory diseases. They were constrained by small sample sizes, use of cross-sectional study design, relying on self-reported primary cooking fuel or stove types for exposure classification, (Clark et al., 2013, Kurmi et al., 2010) or assessment of intermediate traits (e.g. blood pressure) rather than incident diseases. (Fatmi and Coggon, 2016, Arku et al., 2018, Baumgartner et al., 2018) Findings from the limited number of prospective cohort studies that examined cardiovascular mortality have been inconsistent, (Kim et al., 2016, Mitter et al., 2016, Yu et al., 2018, Hystad et al., 2019) and few studies on respiratory diseases exist. (Chan et al., 2019)

Given the “double burden” of exposures to both AAP and HAP in many populations, it is important to assess the health effects of both exposures simultaneously in the same study, using robust quantitative personal exposure data. We conducted a feasibility study to collect and integrate detailed, multi-dimensional AAP and HAP data to enhance personal air pollution exposure characterisation in a large contemporary cohort in China, the China Kadoorie Biobank (CKB). (Chen et al., 2011, Chen et al., 2005) This report describes the design, major procedures and early findings on fuel use, time-activity and air pollution exposure patterns in the study.

## 2 Materials and methods

### 2.1 Study population

The present study population was drawn from the participants in CKB and the details of the CKB design and participant characteristics have been described elsewhere. (Chen et al., 2011, Chen et al., 2005) Briefly, in 2004–2008, ~512,000 adults aged 30–79 years were recruited from five rural and five urban areas across China (Figure A1). Upon recruitment, trained health workers obtained informed consent, undertook physical measurements (e.g. exhaled carbon monoxide [COex]) and administered an electronic questionnaire assessing a range of characteristics (socioeconomic, lifestyle, environment, medical history), including long-term fuel use behaviour as described previously. (Chan et al., 2017) Periodic resurveys are being undertaken in ~ 5% randomly selected subset of surviving participants every 4–5 years since baseline, to repeat the baseline assessments and collect additional data for enhancement. Participants are being followed-up indefinitely since baseline, for fatal and non-fatal events via linkages to established death and disease registries and national health insurance databases.

### 2.2 Sampling and participant recruitment

The present study included two rural (Gansu and Henan) and one urban (Suzhou) of the ten CKB sites, purposively selected to capture a variety of fuel types (gas, electricity, wood and coal) being used for cooking or heating in typical rural and urban settings in China. The planned sample size was 450 participants (150 per site) selected using multi-stage cluster sampling. Considering the decline in solid fuel use in China in the past decade, in each of the sites, one or two of the largest villages (for rural sites) or street communities (for urban sites) with the highest prevalence of solid fuel use at baseline were selected in order to recruit sufficiently large numbers of solid fuel users. To be eligible for invitation, individuals had to be: (i) CKB participants aged < 70 years at the time of recruitment, (ii) living in their current address for at least one year before recruitment, and (iii) without major disability. Individuals who were older or had major disability were excluded because their exposure profiles are likely to be distinct from an average CKB participant.

In order to explore the seasonal variation in fuel use behaviours and related exposures, the fieldwork covered warm (May – September 2017) and cool (November 2017 – January 2018; usual local monthly average temperature < 10 °C) months. Each data collection window consisted of (i) air pollution exposure assessment; (ii) time-activity questionnaire; and (iii) measurement of biomarkers (exhaled carbon monoxide, heart rate and blood oxygen saturation). A household questionnaire was included in the cool season to collect self-reported information on socioeconomic, lifestyle and HAP-related exposure.

**Fig. 1:**
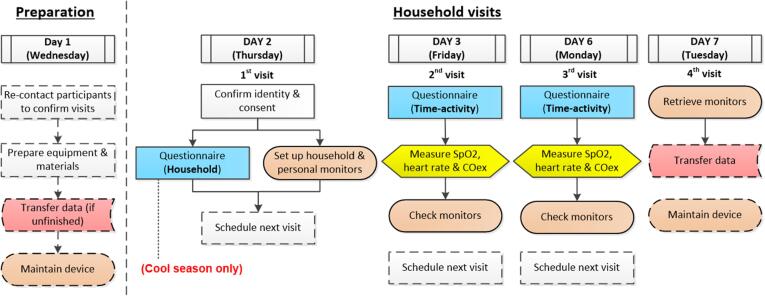
Fieldwork workflow each week. SpO_2_: blood oxygen saturation; COex: exhaled carbon monoxide. See supplementary methods for a brief description on the procedures taken to measure the biomarkers of interest.

Air pollution assessment was done at personal, household and ambient levels. All personal and household measurements were conducted over five consecutive days (120 h) covering both weekdays and weekends, whereas ambient measurements were done throughout the fieldwork period in each study site (4–5 weeks), repeatedly for two seasons. Fig. 1 shows the weekly workflow of the fieldwork.

In each study site, about 40 participants were recruited each week, and the fieldwork ended within five weeks. Overall, a total of 638 participants were invited and 451 (70.7%) participated and provided informed consent in the warm season and were invited for a repeated assessment in the cool season. Thirty-seven warm season participants were unavailable for the cool season campaign and were replaced by other eligible CKB participants from the same community, so 488 individuals were surveyed at least in one season. The original baseline (2004–2008) characteristics of the participants included in the two seasons did not differ significantly (Table B1). The number of participants completing the time-activity questionnaires in the two seasons are presented in Table B2. Ethical approvals were obtained from the Oxford University Tropical Research Ethics Committee, Oxford, UK and the institutional review board of Fuwai Hospital, Chinese Academy of Medical Sciences, Beijing, China.

### 2.3 Data collection

#### 2.3.1 Personal and static household monitoring

We used PATS+CO (Particle and Temperature Sensor plus Carbon Monoxide Sensor; Berkeley Air Monitoring Group, CA, USA), a low cost, light-weighted (110 g) real-time sensor of PM_2.5_ (μg/m^3^) and CO (ppm) concentration, temperature (°C), and relative humidity (RH, %). (Pillarisetti et al., 2017) The PATS+CO is tailored for high intensity HAP measurement and has separate light-scattering (PM_2.5_ detection range: 10–30,000 µg/m^3^) and metal oxide (CO detection range: 0–500 ppm) sensors. For personal monitoring the PATS+CO (and a small external battery weighing 140 g) was attached to a cross body harness or waist belt. The participants were asked to wear the sensor for five consecutive days (typically Thursday–Tuesday) except during bathing and sleeping, when the device should be placed within 1 m of the participant. In each household, another two PATS+CO sensors (also with external batteries) were placed in the kitchen and living room, respectively, at a height of 1.5 m and ≥ 1 m away from any doors or other openings in the walls. The PATS+CO in the kitchen was located ≤ 1 m from the edge of the most frequently used cookstove. At the end of the monitoring period, all PATS+CO sensors were recalibrated in the zeroing box before data being downloaded onto the computer.

#### 2.3.2 Ambient monitoring and residential geocoding

To quantify the background AAP level in local community two Nano Air Stations (NAS-AF100; Sapiens Environmental Technology, Hong Kong, China) were placed in a central location of the community, at least two stories above ground, where no obvious sources of pollution were found, for at least the whole duration of which HAP sampling was implemented in that community. NAS-AF100 measured particulate (PM_1_, PM_2.5_, PM_10_) and gaseous pollutants (CO, O_3_, NO and NO_2_), temperature and RH at a logging interval of 1-minute. The data were wirelessly transmitted to a secured cloud-based server using GSM network.

#### 2.3.3 Quality control and device calibration

PATS+CO has been validated against gravimetric samples in both laboratory and real-world settings internationally, with R^2^ ranging from 0.90 to 0.99 (Pillarisetti et al., 2017). All our devices were factory-calibrated against wood smoke by the manufacturer. Before the commencement of fieldwork, they were calibrated along with filter-based samplers for PM-based measurement, including a personal air sampling pump (SKC Ltd., Dorset, UK) and a MiniVol portable air sampler (Airmetrics, OR, USA) (see Appendix C. Supplementary Methods for a brief description) and 60 ppm CO gas following a standardised protocol. We have also conducted side-by-side inter-comparison tests in a laboratory environment following standardised experimental procedures (Pillarisetti et al., 2017), and confirmed data comparability across PATS+CO devices (Pearson correlation coefficients: 0.85 to 0.99). Prior to any data collection (including laboratory experiment and fieldwork) all devices were allowed to warm-up for 24–48 h to reach a steady state. For deployment, the PATS+COs were initialised and calibrated in a “zeroing box” (filled with HEPA-filtered air) for 10 min before being distributed. The time-resolved PM_2.5_ concentration data are derived from particle counts from the photometer response with internal calibration for real-time temperature and RH variations.

All NAS-AF100 were laboratory-calibrated before deployment. Gravimetric sampling for device calibration was done following established procedures described previously (see Appendix C. Supplementary Methods for further details) (Tong et al., 2018, Cao et al., 2005). The monitors were returned to the manufacturer for re-calibration at the end of each data collection campaign, following procedures described elsewhere (Wei et al., 2018, Wei et al., 2020). The longitude and latitude of the participants’ residences were obtained using a commercial GPS locator (ICEGPS, Shenzhen, China) and documented during the first household visit when the sensors were deployed.

### 2.4 Questionnaires

The household questionnaire collected data on socioeconomic, lifestyle and HAP-related factors, including household cooking frequency and all fuel types in use for cooking and heating in the household. For each reported fuel type, the frequency (for cooking: “most meals” versus “sometimes”) or duration (for heating: hours of use for heating on a typical day) of use and number of years of use throughout their life were recorded.

All participants were asked to recall their activities during the past 24 h twice, first on the Friday (recall for Thursday) and second on the Monday (recall for Sunday) of the 5-day monitoring period (Fig. 1). The time-activity questionnaire was designed to capture the type, location and length of time spent on each distinctive activity and whether the participants smoked or were exposed to environmental tobacco smoke (ETS) during each activity reported. The visit schedule was designed to capture the potential variability of time spent on activities on weekdays and weekends.

### 2.5 Data analysis

Percentages and means of selected background and HAP-related characteristics were compared across the three study sites. Regular cooking was defined as cooking for at least a few times a week as in our previous studies (Yu et al., 2018, Chan et al., 2019a, Chan et al., 2019b). A composite variable of cooking fuel combination was derived based on the types of fuels used for most meals (or all reported fuel types if none was noted as used for most meals). Similarly, a composite variable of heating fuel combination was derived from the fuel type used for the longest duration (hour) on a typical day that required heating. The two composite variables were restricted to individuals whose household cooked or used heating regularly, respectively.

The time-activity questionnaire provided data on frequency and duration of cooking, duration of heating, frequency of smoking and ETS exposure, time spent on different activities and locations. These were compared across the weekday and weekend visits, and between the two seasons. The total duration and proportion of recorded duration (total and waking time) during which the participants were wearing the personal PATS+CO were also computed to assess the self-reported compliance of wearing the monitor.

For the device data, initially, the PATS+CO data from 10% randomly selected participants were inspected graphically on time-series plots to understand the diversity and complexity of the data. Then, a data processing protocol was developed to remove potentially erroneous or unreliable data before analysis. This report focuses on the PM_2.5_ data recorded in PATS+CO. First, we removed data recorded during the initial and final 60 min of the monitoring period (when the household visit for device set up and collection occurred), as these data do not reflect the participants’ usual exposure patterns. Then, datasets with < 24 h’ worth of data (likely due to battery failure) and those with persistently high or low PM_2.5_ levels for > 50% of the monitoring period were removed to reduce potential bias. A moving-median smoothing method was applied to remove sporadic extreme spikes that could happen due to physical shock or direct sunlight interference to the nephelometer. Similar procedures were applied to ambient PM_2.5_ data from NAS-AF100. For this report, the time series data were compressed (averaged) to produce 24-hour time-series plots by device location (personal, kitchen, living room and outdoor) across seasons and study sites, and the corresponding overall means and standard deviations (SDs) were compared.

## 3 Results

### 3.1 Participant characteristics

Of the 449 participants who completed the household questionnaire in the cool season, the mean [SD] age was 58.0 [6.8] years and 72% were females. Participants from urban Suzhou were slightly older, less likely to be female, had substantially higher household income, and none of them were agricultural workers while the majority of those from rural Gansu and Henan were either agricultural workers or home-makers (Table 1). Current-regular smoking prevalence was low (~15%, 64/65 were men), but exposure to ETS was common (42% for 6–7 days/ week).

**Table 1:** Background characteristics of study participants by study area.

Characteristics	Suzhou (Urban)	Gansu (Rural)	Henan (Rural)	Overall
**Age - years, mean (SD)**	59.8 (7.0)	57.4 (7.3)	56.8 (5.6)	58.0 (6.8)
**Female**	103 (69.1)	110 (73.3)	112 (74.7)	325 (72.4)
**Annual household income – Yuan, n (%)**				
<35,000	2 (1.3)	63 (42.0)	100 (66.7)	165 (36.8)
35,000–74,999	21 (14.1)	80 (53.3)	42 (28.0)	143 (31.9)
≥75,000	126 (84.5)	7 (4.7)	8 (5.3)	141 (31.4)
**Occupation, n (%)**				
Agricultural worker	0 (0.0)	67 (44.7)	101 (67.3)	168 (37.4)
Factory worker	16 (10.7)	0 (0.0)	6 (4.0)	22 (4.9)
Non-manual labour	6 (4.0)	1 (0.7)	5 (3.3)	12 (2.7)
Retired	99 (66.4)	2 (1.3)	0 (0.0)	101 (22.5)
Home-maker	15 (10.1)	79 (52.7)	34 (22.7)	128 (28.5)
Self/un-employed or other	13 (8.7)	1 (0.7)	4 (2.7)	18 (4.0)
**Type of dwelling, n (%)**				
Apartment	16 (10.7)	0 (0.0)	0 (0.0)	16 (3.6)
House	133 (89.3)	150 (100.0)	150 (100.0)	433 (96.4)
**Smoking, n (%)**				
Do not smoke now	122 (81.9)	122 (81.3)	129 (86.0)	373 (83.1)
Occasional	4 (2.7)	2 (1.3)	5 (3.3)	11 (2.5)
Current-regular	23 (15.4)	26 (17.4)	16 (10.7)	65 (14.5)
Frequency of ETS* exposure, n (%)				
Never	75 (51.0)	30 (20.4)	45 (30.6)	150 (34.0)
<6 day/ week	12 (8.1)	49 (33.3)	46 (31.3)	107 (24.3)
6–7 day/ week	60 (40.8)	68 (46.3)	56 (38.1)	184 (41.7)

*ETS: environmental tobacco smoke.

### 3.2 Cooking, heating and ventilation

The majority (76%) of the participants cooked weekly or daily (mean cooking time = 2.7 (SD 0.7) hours/ day) and the rest cooked a few times a month or less, but about half of them lived in households where cooking was regularly practised (Table 2). Of participants who lived in regular-cooking households, >60% of those from Suzhou reported using only one fuel type, whereas most households from Gansu (90%) and Henan (95%) used two to three types of fuels. Eighty-nine participants (23%) reported that fuels were used “sometimes” only (i.e. no fuel used for “most meals”), with the majority used clean fuels only (n = 79, mostly from Suzhou). Among the remaining participants, the most common combination was clean fuels only (51%), followed by clean fuels and wood (21%, all in Gansu) and clean fuels and coal (15%, mostly in Henan). Most participants from Suzhou had a ventilated kitchen (98%), but it was less common in Gansu (70%) and Henan (45%). The majority of those from Gansu (93%) and Henan (69%) always opened their windows when cooking, but this was less common in Suzhou (61%). About 12% of all participants (mostly from Gansu) reported that their kitchen always became smoky while cooking.

**Table 2:** Cooking- and heating-related characteristics of study participants by study area.

Characteristics	Suzhou (Urban)	Gansu (Rural)	Henan (Rural)	Overall
**Cooking exposure, n (%)**				
Personal regular	108 (72.5)	117 (78.0)	117 (78.0)	342 (76.2)
Household regular, personal non-regular	23 (15.4)	21 (14.0)	9 (6.0)	53 (11.8)
Non-regular	18 (12.1)	12 (8.0)	24 (16.0)	54 (12.0)
Cooking duration - hours, mean (SD)*	2.7 (0.7)	2.8 (0.8)	2.5 (0.7)	2.7 (0.7)
Number of cooking fuel reported, median (IQR)*	1 (1–2)	3 (2–3)	2 (2–3)	2 (2–3)
Cooking fuel combination for most meals, n (%)*				
All reported fuel(s) was used sometimes	66 (50.4)	8 (5.8)	15 (11.9)	89 (22.5)
Clean fuels only	64 (48.9)	48 (34.8)	43 (34.1)	155 (39.2)
Clean fuels and coal	0 (0)	11 (8.0)	35 (27.8)	46 (11.7)
Clean fuels and mixed solid fuels	0 (0)	5 (3.6)	0 (0)	5 (1.3)
Clean fuels and wood	0 (0)	64 (46.4)	0 (0)	64 (16.2)
Coal only	0 (0)	1 (0.7)	33 (26.2)	34 (8.6)
Mixed solid fuels	0 (0)	1 (0.7)	0 (0)	1 (0.3)
Wood only	1 (0.8)	0 (0)	0 (0)	1 (0.3)
Cooking fuel in those reported all fuels were used sometimes, n (%)*^,^†				
Clean fuels	66 (1 0 0)	6 (75.0)	7 (46.7)	79 (88.8)
Clean fuels and coal	0 (0)	0 (0)	8 (53.3)	8 (9.0)
Clean fuels and wood	0 (0)	2 (25.0)	0 (0)	2 (2.3)
Ventilated kitchen, n(%)*	126 (98.4)	96 (70.1)	58 (45.0)	280 (71.1)
Windows opened when cooking, n (%)*				
Always	78 (60.9)	128 (93.4)	89 (69.0)	295 (74.9)
Sometimes	17 (13.3)	8 (5.8)	22 (17.1)	47 (11.9)
Rarely/ never/ no window in the kitchen	33 (25.8)	1 (0.7)	18 (14.0)	52 (13.2)
Smoky kitchen when cooking, n (%)*				
Always	1 (0.8)	38 (27.7)	7 (5.4)	46 (11.7)
Sometimes	6 (4.7)	68 (49.6)	31 (24.0)	105 (26.7)
Rarely/ never	121 (94.5)	31 (22.6)	91 (70.5)	243 (61.7)
**Heating frequency, n (%)**				
Daily or almost every day	25 (16.8)	136 (90.7)	111 (74.0)	272 (60.6)
A few times a week	10 (6.7)	0 (0.0)	3 (2.0)	13 (2.9)
A few times a month	8 (5.4)	0 (0.0)	3 (2.0)	11 (2.5)
No heating	106 (71.1)	14 (9.3)	33 (22.0)	153 (34.1)
**Reason of no heating, n (%)**				
No such need	106 (100.0)	12 (85.7)	29 (87.9)	147 (96.1)
Cannot afford	0 (0.0)	1 (7.1)	0 (0.0)	1 (0.7)
Inconvenience	0 (0.0)	1 (7.1)	4 (12.1)	5 (3.3)
**Typical heating duration** - hours**, median (IQR)**‡	3 (2–3)	24 (24–24)	24 (24–24)	
**Presence of central heating system, n (%)**‡	1 (2.3)	6 (4.4)	5 (4.3)	12 (4.1)
**Number of heating fuel reported, median (IQR)**‡	1 (1–1)	1 (1–2)	1 (1–1)	1 (1–1)
**Heating fuel combination (used for longest hour), n (%)**‡				
Clean fuels only	43 (1 0 0)	3 (2.2)	6 (5.1)	52 (17.6)
Coal only	0 (0)	62 (45.6)	100 (85.5)	162 (54.7)
Charcoal only	0 (0)	0 (0)	9 (7.7)	9 (3.0)
Wood only	0 (0)	48 (35.3)	0 (0)	48 (16.2)
Mixed solid fuels	0 (0)	23 (16.9)	0 (0)	23 (7.8)
Others	0 (0)	0 (0)	2 (1.7)	2 (0.7)
**Ventilated heat-stove, n(%)**‡	9 (20.9)	133 (97.8)	107 (91.5)	237 (84.1)
**Smoky home while heating, n (%)**‡				
Always	0 (0.0)	1 (0.7)	0 (0.0)	1 (0.3)
Sometimes	0 (0.0)	111 (81.6)	2 (1.7)	113 (38.2)
Rarely/ never	43 (100.0)	24 (17.7)	115 (98.3)	182 (61.5)

* Percentage counted only among participants who live in households where regular cooking occurred.

† Reported as fuel used for cooking sometimes.

‡ Percentage counted only among those reported using heating for at least a few times a month.

Winter heating was prevalent in Gansu (91% daily) and Henan (74% daily) but not in Suzhou (17% daily) (Table 2), mainly because it was not considered necessary (96%). Most of the participants from Gansu (97%) and Henan (92%) who used heating in winter reported whole-day heating, whereas 81% of those from Suzhou heated their home for up to three hours only. Multiple fuel use for heating was uncommon, with 78% reported using only one fuel type. Among heating fuel users, all those from Suzhou used clean fuels, those from Henan primarily used coal (86%) and those from Gansu used either coal (46%), wood (35%) or a mix of solid fuels (17%). Most heat-stoves used in Gansu and Henan were ventilated (>90%), unlike in Suzhou where 79% had no ventilation. Smoky home while heating was only common in Gansu (82%) but not in other areas.

### 3.3 Time-activity patterns

Overall, participants tended to spend the majority of their time at home (median = 20.2–21.5 h/ recall day) and indoors (i.e. home, indoor workplace or public spaces; median = 21.5–23.0 h/ recall day), especially in the cool season (Tables 3 and B3). The cooking frequency and duration in the weekday and weekend assessments were largely similar within each season, but cooking was practiced more frequently (73% versus 56% ≥ twice/ day) and for longer total duration (median 2.5 [IQR 1.7–3.0] versus 1.7 [1.0–2.5] hours) in the cool than in the warm season.

While ~15% of the participants (men 50%, women 0.3%) reported current-regular smoking in the household questionnaire (Table 1), ~10% (men 47%, women 0%) reported smoking during the time-activity recall period in both seasons (Table 3). About 15% (men 8%, women 18%) of participants in the warm and cool season were exposed to ETS. The reported duration of carrying the personal PATS+CO were largely similar in both seasons (median = 12.5 h; up to 83% awake time), but weekend compliance was consistently higher than weekday.

**Table 3:** Selected time-activity characteristics by weekday and weekend household visit in warm and cool season.

	**Warm season**			**Cool season**		
**Characteristics assessed in the day recalled**	**Weekday (n = 450)**	**Weekend (n = 448)**	**Overall (n = 898)**	**Weekday (n = 449)**	**Weekend (n = 448)**	**Overall (n = 897)**
Hours spent at different locations or activities, median (IQR)*						
At home	20.3 (16.2–23.3)	20.2 (16.5–23.6)	20.3 (16.5–23.5)	21.5 (18.3–24.0)	21.0 (18.3–24.0)	21.3 (18.3–24.0)
Indoors	21.5 (18.0–23.8)	21.8 (19.0–24.0)	21.7 (18.5–24.0)	23.0 (20.5–24.0)	23.0 (20.0–24.0)	23.0 (20.0–24.0)
Roadside	2.0 (1.0–4.0)	2.0 (1.0–4.0)	2.0 (1.0–4.0)	2.0 (1.0–3.7)	1.8 (1.0–3.0)	2.0 (1.0–3.3)
Cooking	1.6 (1.0–2.3)	1.8 (1.0–2.5)	1.7 (1.0–2.5)	2.5 (1.5–3.0)	2.5 (1.8–3.0)	2.5 (1.7–3.0)
Heating	NA	24.2 (24.0–24.5)	24.2 (24.0–24.5)	24.0 (24.0–24.5)	24.0 (24.0–24.5)	24.0 (24.0–24.5)
**Cooking frequency, n (%)**						
0	121 (26.9)	123 (27.5)	244 (27.2)	89 (19.8)	83 (18.5)	172 (19.2)
Once	82 (18.2)	59 (13.2)	141 (15.7)	33 (7.4)	36 (8.0)	69 (7.7)
2 times	139 (30.9)	152 (33.9)	291 (32.4)	117 (26.1)	111 (24.8)	228 (25.4)
≥ 3 times	108 (24.0)	114 (25.5)	222 (24.7)	210 (46.8)	218 (48.7)	428 (47.7)
**Smoking frequency, n (%)**†						
0	406 (90.2)	402 (89.7)	808 (90.0)	402 (89.5)	403 (90.0)	805 (89.7)
1–5 times	23 (5.1)	28 (6.3)	51 (5.7)	31 (6.9)	28 (6.3)	59 (6.6)
≥ 6 times	21 (4.7)	18 (4.0)	39 (4.3)	16 (3.6)	17 (3.8)	33 (3.7)
**ETS exposure frequency, n (%)**‡						
0	383 (85.1)	371 (82.8)	754 (84.0)	380 (84.6)	388 (86.6)	768 (85.6)
1–4 times	43 (9.6)	54 (12.1)	97 (10.8)	37 (8.2)	39 (8.7)	76 (8.5)
≥ 5 times	24 (5.3)	23 (5.1)	47 (5.2)	32 (7.1)	21 (4.7)	53 (5.9)
PATS+CO compliance*						
**Duration wearing PATS+CO - hours, median (IQR)**	12.0 (9.3–14.2)	13.0 (10.2–15.0)	12.5 (9.5–14.7)	12.0 (9.9–14.0)	13.2 (11.0–15.0)	12.5 (10.2–14.5)
**% of awake time wearing PATS+CO, median (IQR)**	79.0 (60.4–93.5)	86.7 (49.3–100)	81.8 (56.7–100)	80.8 (61.8–93.3)	86.4 (57.1–100)	83.0 (60.0–100)

* Median (inter-quartile range [IQR]) only among those who reported activities in relevant locations or practised the activity during the designated recall period.

† Median frequency in those who have smoked at least once during the day of assessment = 5 times.

‡ ETS: environmental tobacco smoke; median frequency in those who was exposed to ETS during the day of assessment = 4 times.

### 3.4 Device data

**Fig. 2:**
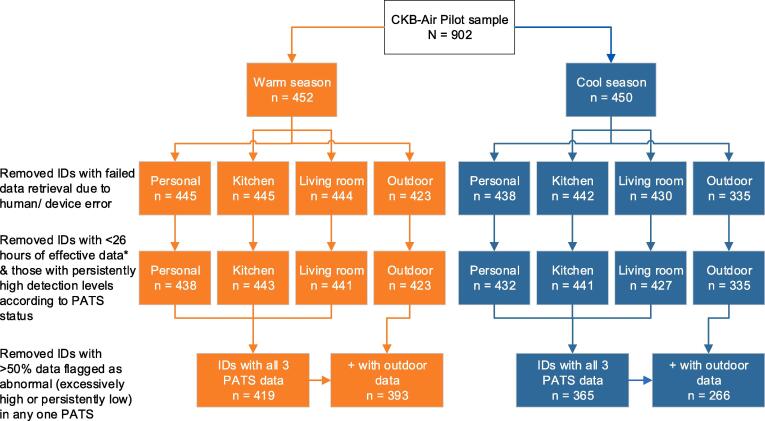
Flowchart of PATS+CO data exclusion by season and device location. *Cut-off designed to ensure that there was at least 24 h’ worth of data after excluding the first and last hour from the time series (to remove data likely to be influenced by the initial device deployment and final device collection work.

After data cleaning, 419 (92.7%) and 365 (81.1%) participants had satisfactory PM_2.5_ data from all three PATS+COs in warm and cool seasons, respectively (Fig. 2). A smaller number of them (warm season: 393 [86.9%]; cool season: 266 [59.1%]) also had concurrent data from AAP monitors, largely due to delayed device deployment (particularly in the cool season), but also power outage and memory loss.

**Fig. 3:**
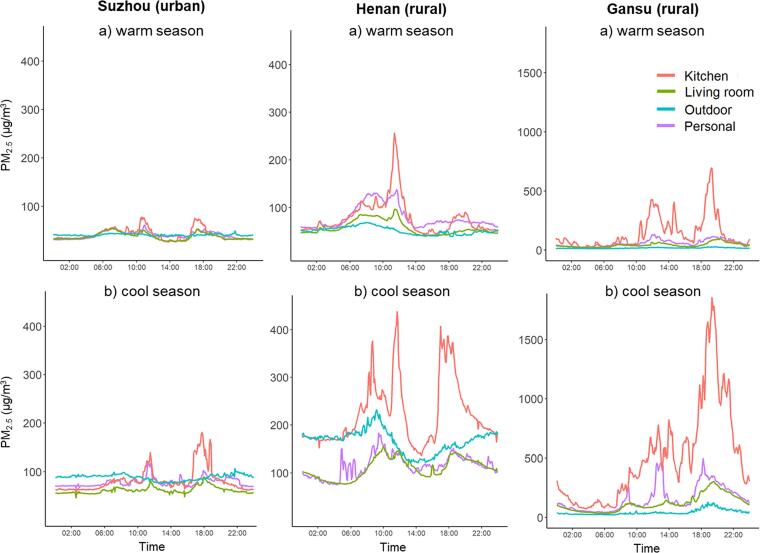
Averaged 24-hour variation of PM_2.5_ levels recorded in the personal, kitchen, living room, and ambient monitors across the three study sites in the warm and cool season. Time frame displayed: from 00:00 to 24:00 on the x-axes.

In both seasons, the PM_2.5_ levels were generally the highest in Gansu, followed by Henan and Suzhou (Fig. 3 **and** Table 4). The exposure was substantially (2–3 times) higher in the cool than warm season for all device locations across all study sites. The temporal variation at the four locations are broadly in line with each other, with prominent peaks commonly seen in the kitchen in the early morning and around noon and evening times, when people typically cook (as consistently reported in the time-activity questionnaire). Notably, the exposure in the cool season tend to remain at a high level (>100 µg/m^3^) even out of the typical cooking times.

**Table 4:** Means and standard deviation (SD) of fine particulate matter (PM_2.5_) concentration (µg/m^3^) by season and study area.

	Personal		Kitchen		Living room		Outdoor	
	Mean	SD	Mean	SD	Mean	SD	Mean	SD
**Warm season**								
**Suzhou (urban)**	40.7	81.1	41.6	103.2	37.9	65.4	40.9	21.3
**Gansu (rural)**	60.4	269.9	142.3	1415.1	43.4	130.3	17.9	9.6
**Henan (rural)**	78.6	145.9	77.5	206.6	57.4	69.7	51.5	23.0
**Cool season**								
**Suzhou (urban)**	78.2	293.1	81.6	270.2	61.9	89.4	88.5	51.0
**Gansu (rural)**	160.5	1514.9	508.1	4195.6	118.3	359.0	44.3	32.2
**Henan (rural)**	114.8	329.5	222.3	868.1	109.1	165.0	166.4	105.1

PM_2.5_ levels recorded in the kitchen tend to be markedly higher than other device locations in the rural sites but less so in Suzhou, where similar levels were observed across all locations in the warm season (~40 µg/m^3^), and outdoor levels were considerably higher than the indoor locations in the cool season (mean = 88.5 µg/m^3^). Interestingly, in Henan, outdoor PM_2.5_ levels were the lowest across the four locations in the warm season but came just below the kitchen in the cool season; whereas the corresponding outdoor levels in Gansu were consistently the lowest.

When stratified by sex, the mean personal PM_2.5_ levels recorded in women appeared broadly similar to those of men in Gansu (mean = 61.7 versus 57.0 µg/m^3^) and Henan (mean = 80.3 versus 73.3 µg/m^3^) but considerably lower in Suzhou in the warm season (mean = 36.6 versus 50.9 µg/m^3^); whereas the levels in women in winter appeared either higher (mean_Suzhou_ = 91.9 versus 45.1 µg/m^3^; mean_Gansu_ = 171.3 versus 129.7 µg/m^3^) or similar (mean_Henan_ = 114.9 versus 114.4 µg/m^3^) (Table B.4). The time-resolved exposure patterns were broadly similar in shape by sex, but women tended to have longer elevated exposure periods especially during typical cooking times, whereas men tended to have more irregular, short-duration spikes (Figures A.2 and A.3).

## 4 Discussion

We reported the design, procedures and initial findings of a pilot study involving extensive air pollution measurements and questionnaire assessments in 488 individuals recruited from two rural and one urban site of China. Although it is primarily a feasibility study, the data collected offer valuable insight into a range of factors associated with air pollution exposure, which would in turn inform the modelling strategies for personal exposure and the design of assessment tools for future cohort studies of greater scale.

### 4.1 Fuel use and ventilation

Most previous epidemiological studies on HAP recorded only primary fuel types, with many classifying non-cooking individuals as “unexposed” (Kurmi et al., 2010, Fatmi and Coggon, 2016, Kim et al., 2016, Mitter et al., 2016, Sana et al., 2018, Jary et al., 2016). In CKB-Air, about half of the non-cooking participants were “exposed” to regular cooking in the household, highlighting the potential bias of considering them as the unexposed group (Sidhu et al., 2017, Li et al., 2016). We also revealed a fuel-stacking phenomenon alongside increased clean fuel use (compared with previous CKB data (Chan et al., 2017) in line with a few recent Chinese studies (Ni et al., 2016, Tao et al., 2018). Fuel-stacking in CKB-Air was exclusive to rural participants, who were still undergoing fuel transition. As secondary use of solid fuels could result in significantly elevated HAP exposure, our findings underline the need of routinely collecting comprehensive fuel use data in future HAP studies. Notably, fuel-stacking was much less common for heating compared to cooking and the prevalence of solid fuel use for heating remained high among rural participants. This corroborates with our previous observations that heating fuel modernised more slowly than cooking fuels (Chan et al., 2017), and it could be an under-recognised source of HAP in China and other LMICs (Chen et al., 2018).

Participants from two rural areas used heating in winter much more frequently and for longer duration than those from the urban area. The main reason for not having heating was no perceived need, which is reasonable to expect in Suzhou due to the relatively short and mild winters (only 10/156 months with average ambient temperature ≤ 5 °C during 2005–2017 (China Meteorological Data Service Center. China Meteorological Data Service Center, 2018). However, it is surprising to see that 10–20% of participants from Gansu (45/156 months ≤ 5 °C during 2005–2017) and Henan (31/156 months) felt heating was unnecessary. Perceived need of heating has rarely been assessed in epidemiological studies, but our observations lead to a key question as to whether the perceived need of heating matches with the actual need in coping with cold temperatures and the associated health risks (Gasparrini et al., 2015, Lewington et al., 2012, Saeki et al., 2014).

The site-specific prevalence of ventilated kitchen in CKB-Air was comparable to those observed in the second resurvey (2013–2014) of CKB, coinciding with the previously described relatively stagnant trend of ventilation adoption (Chan et al., 2017). Interestingly, self-reported smoky kitchen was particularly common in Gansu where the kitchen PM_2.5_ levels were the highest, even though most people kept their kitchen windows open (which was supposed to improve ventilation). This highlights the complexity of the practice and effectiveness of mitigation behaviours against air pollution (HAP in this case), which have been commonly overlooked in previous epidemiological studies.

### 4.2 Time-activity patterns

Conventional AAP exposure classification methods rely chiefly on residential addresses (Brauer et al., 2016), and one prevailing criticism has been the inability to account for intra- and inter-personal variability of time-activity patterns (e.g. exposure in locations distant from individuals’ residential addresses) (Han and Naeher, 2006, Qi et al., 2018). In this sample of older adults, participants spent up to 90% of their time indoors. This is broadly consistent with previous national data from the United States (Klepeis et al., 2001) and China (Duan et al., 2015), but our participants spent much greater proportion of their indoor time at home, possibly because of the relatively old age and high proportion of home-makers (29%) and retirees (23%). The high proportion of time spent at home means that the conventional residence-based AAP exposure assessment may be subject to less misclassification in older adults in settings where AAP is the primary source of personal air pollution exposure (e.g. urban cities). However, in solid fuel-reliant communities, exposure to HAP must be taken into account, ideally with direct measurements or adjustment factors based on fuel use patterns – but this has been rarely accounted for in previous AAP studies.

There has been some evidence suggesting solid fuel use for heating to be a primary contributor to the significantly higher HAP levels in cool season, (Chen et al., 2018, Chen et al., 2016) but little is known about the extent to which the seasonal variability of time-activity patterns, particularly changes in cooking behaviour, may also contribute to such differences. The present study showed that participants tended to cook more frequently and for longer duration in cool (mean 2.5 h) than in warm (1.7 h) season. It is reassuring that the average cooking duration ascertained via the time-activity questionnaire in the cool season was largely similar to what was reported for the household questionnaire.

### 4.3 Direct measurement data

Several recent studies focussing on HAP in LMICs have been undertaken to directly measure personal exposure to PM_2.5_ within a cohort study to develop personal exposure models for epidemiological analysis. (Arku et al., 2018, Tonne et al., 2017, Balakrishnan et al., 2015) Most of these studies involved primarily microenvironment monitoring supplemented by personal exposure measurements in a smaller subset, using time-integrated monitors operating over relatively short assessment periods (24–48 h). Comparatively, our study adopted a more intensive and extensive measurement protocol, collecting personal, household (living room and kitchen) and ambient exposure data for most participants for ~ 120 consecutive hours (covering weekdays and weekends) for two seasons. This provides detailed data for in-depth investigation on the intra- and inter-personal variability of air pollution exposure, as well as the relationship between personal, household and ambient levels of PM_2.5_. Although the main device (PATS+CO) we used has no gravimetric sampling functionality, it has been carefully validated and used extensively in field-based measurement studies, and it offers time-resolved data that enables fine-mapping of exposure levels to different activities (e.g. cooking).

The gradient of average PM_2.5_ exposure across the study sites is consistent with the regional fuel use patterns, with substantially higher levels recorded in the two rural sites, where solid fuels were more commonly used for cooking and heating, than in Suzhou, where virtually all participants used clean fuels. Between the two rural sites, Gansu had significantly higher PM_2.5_ exposure, particularly in the kitchen (Gansu versus Henan: mean_warm_season_ = 142.3 versus 77.5 µg/m^3^; mean_cool_season_ = 508.1 versus 222.3 µg/m^3^), consistent with the fact that more participants from Gansu used wood, which is known to be associated with higher PM_2.5_ emission than coal (commonly used in Henan). (Clark et al., 2013) Given the vast heterogeneity of fuel use characteristics and challenges in conducting large-scale exposure measurement studies, there exist no reliable global or national (for China) estimates of PM_2.5_ exposure related to any specific fuel types. (Clark et al., 2013) Nonetheless, a few previous studies conducted in rural China reported broadly similar indoor PM_2.5_ levels in communities where wood (mean_summer_ 110–150 µg/m^3^; mean_winter_ = 240–500 µg/m^3^) (Carter et al., 2016, Snider et al., 2018) or coal (mean_summer/winter_ = 100–200 µg/m^3^) (Hu et al., 2014) use for cooking and/or heating were common. In urban Suzhou, the average PM_2.5_ exposure across the four device locations were largely similar in the warm season (~40 µg/m^3^), but that in the outdoor environment was distinctly higher in the cool season (~90 µg/m^3^), suggesting that ambient sources play a more important role during winter months in this area. This is again consistent with recent estimates in Chinese cities, where ambient PM_2.5_ levels have been much higher in winter (~90–120 µg/m^3^) than in summer (~30–50 µg/m^3^) (Shen et al., 2016, Huang et al., 2020, Wang et al., 2020) possibly due to regional air pollution associated with increased demand for heating and temperature inversion effect. Interestingly, despite exclusive clean fuel usage in Suzhou, on average two peaks of PM_2.5_ were detected during typical cooking times in the kitchen, with milder but similar peaks registered in the personal monitors. This might reflect the cooking fumes associated with Chinese style cooking, which often involve high-temperature stir-frying. (Li et al., 2015, Wang et al., 2015)

Similar but markedly more extreme peaks consistent with solid fuel use for cooking were observed in kitchens in Gansu and Henan. While it is typically expected that ambient PM_2.5_ contributes a smaller proportion of the total personal exposure in solid fuel-reliant communities (as in Gansu for both seasons and in Henan in the warm season), ambient PM_2.5_ levels in Henan during the cool season were just below those recorded in the kitchen. Both rural sites involved heavy use of solid fuels for heating in the cool season, which could be a major contributor of community-level ambient PM_2.5_. (Snider et al., 2018, Shupler et al., 2018) However, the Gansu site is in a mountainous region with low population density, away from urban settlements and busy highways; whereas the Henan site is a more crowded community < 30 km from an industrial town with heavy road traffic and longstanding regional ambient air pollution problems. These contextual factors may have influenced the dispersion of air pollution in the two study sites.

Notably, about 70% of the study participants were women, who tend to have a dominant role in cooking and are predominantly non-smokers (>97%); whereas men tend to cook less regularly and much more likely to smoke (>60%) in the study population, (Chan et al., 2017) so the overall exposure pattern is likely to be more relevant to women and non-smokers. Generally speaking, the 24-hour exposure patterns were somewhat similar in men and women, with major exposure peaks around typical cooking times and significantly higher PM_2.5_ exposure in the cool season (except for men in Suzhou), which is well expected given the higher background HAP and AAP levels in winter. (Chen et al., 2018, Carter et al., 2016, Snider et al., 2018) The tendency of higher PM_2.5_ exposure in women, especially in the cool season, could be explained by the longer time women spent on cooking or in the kitchen compared to men; whereas the more frequent short-duration spikes in men, especially out of typical cooking or sleeping/ heating times, are consistent with active or passive smoking exposure.

### 4.4 Implications for future studies

The successful completion of our fieldwork to collect detailed and good quality time-resolved air pollution data demonstrates the feasibility and potential value of this type of studies. In particular, the intensive 120-hour air pollution measurements along with repeated household visits for time-activity recall had been well accepted in this study population. Local fieldworkers with little prior experience can be trained rapidly to handle a wide range of data collection instruments including air pollution monitors. The aforementioned new evidence on different behavioural factors reassures the need of collecting detailed fuel use and time-activity data in future studies. The time-solved data presented allude to the complexity of personal air pollution exposure, and its relationships with static environmental measurements or address-based modelling in rapidly transiting economies like China. To investigate this further, work has been planned to associate PM_2.5_ levels (average and peak values) with various activities in different seasons, alongside characteristics reported in the household questionnaire (see Appendix D. Supplementary Discussion for further details).

Our study provides a framework for designing future measurement campaigns to collect personal environmental exposure data in larger and more representative samples in low- and middle-income settings. Further work is also underway to integrate the questionnaire and device data collected and establish pipelines to develop prediction models for personal exposure to PM_2.5_ using established methods. (Chen et al., 2018) By collecting detailed exposure data in a small subset and subsequently building personal exposure prediction models applicable to the entire study population, cost-effectiveness could be achieved. The models developed will also add-value to existing epidemiological studies with only qualitative or semi-quantitative exposure data based on self-reported fuel use to assign personal exposure to HAP and/ or AAP (see Appendix D. Supplementary Discussion for further details).

### 4.5 Limitations

Despite the great details of data being collected to address some key knowledge gaps in the literature, this study has several limitations. Fuel use data were self-reported and semi-quantitative; time-activity were recalled with no objective means of validation (e.g. imageries from wearable cameras); household environment characteristics (e.g. indoor-outdoor air exchange rate, kitchen size) were not measured; and limited health outcome data were collected, although the participants are still being followed-up for health events via record linkages. With the time-resolved PATS+CO as the primary device, we only collected gold standard gravimetric data for a small random subset of participants. There was also considerable loss of air pollution data due to fieldwork or device problems. Furthermore, only ecological comparisons were made in the present manuscript to interpret the PM_2.5_ data in relation to fuel use patterns across the study sites, but further investigation on individual-level data is underway. More in-depth analysis to integrate and cross-reference the time-activity and device data will also be conducted to better understand the temporal variation of PM_2.5_ levels at different device locations, and the inter-location variability. Finally, this study only involved one urban and two rural communities nested within the CKB and recruited a high proportion of women and older adults, so the results are unlikely to be generalisable to a national level or other populations with distinctive environmental characteristics (e.g. fuel use, traffic density). While the lack of representativeness is a common problem in air pollution measurement studies, the overall personal time-activity and PM_2.5_ exposure patterns would be biased towards women, who tend to have a more important role in cooking and heating and rarely smoke in this population.

### 4.6 Summary

We described a study framework designed to efficiently collect in-depth, multi-dimensional data for accurate personal air pollution exposure quantification in a real-world setting in China. The study also provided new evidence supporting the emergence of fuel-stacking behaviour, the significant delay in rate of modernisation of heating fuel and ventilation (compared to cooking fuels), the regional and seasonal variability of time-activity patterns (e.g. cooking frequency and duration) and objectively measured PM_2.5_ concentrations at personal, household and ambient levels. These underscore the limitations of existing epidemiological evidence on the health impact of air pollution and shed light on key areas of methodology improvements for future studies, particularly the need to collect and integrate multi-dimensional personal exposure data.

## CRediT authorship contribution statement

**Ka Hung Chan:** Conceptualization, Methodology, Software, Formal analysis, Investigation, Resources, Data curation, Writing - original draft, Writing - review & editing, Visualization. **Xi Xia:** Software, Formal analysis, Data curation, Visualization, Writing - review & editing. **Kin Fai Ho:** Conceptualization, Methodology, Validation, Data curation, Writing - review & editing. **Yu Guo:** Resources, Project administration. **Om P Kurmi:** Conceptualization, Project administration, Funding acquisition. **Huaidong Du:** Resources, Project administration. **Derrick A Bennett:** Resources, Project administration. **Zheng Bian:** Resources, Project administration. **Haidong Kan:** Methodology, Resources. **John McDonnell:** Software, Data curation. **Dan Schmidt:** Software, Data curation. **Rene Kerosi:** Software, Data curation. **Liming Li:** Resources, Supervision. **Kin Bong Hubert Lam:** Conceptualization, Methodology, Investigation, Resources, Writing - review & editing, Supervision, Project administration, Funding acquisition. **Zhengming Chen:** Conceptualization, Methodology, Supervision, Funding acquisition.

## Declaration of Competing Interest

The authors declare that they have no known competing financial interests or personal relationships that could have appeared to influence the work reported in this paper.
