# Supplementary material for: Regional and seasonal variations in household and personal exposures to air pollution in one urban and two rural Chinese communities: A pilot study to collect time-resolved data using static and wearable devices
Source: Environ Int. 2021 Jan;146:106217. doi: 10.1016/j.envint.2020.106217 (PMC7786640; doi:10.1016/j.envint.2020.106217)
Supplement: Supplementary data 1 [file mmc1.docx]

**Supplementary Appendices**

**Regional and seasonal variations in household and personal exposures to air pollution in one urban and two rural Chinese communities: a pilot study to collect time-resolved data using static and wearable devices**

Ka Hung Chan^1^, Xi Xia^2^, Kin Fai Ho^2^, Yu Guo^3^, Om P Kurmi^4^, Huaidong Du^1,5^, Derrick A Bennett^1^, Zheng Bian^3^, Haidong Kan^6^, Liming Li^7^, Kin Bong Hubert Lam^1*^, Zhengming Chen^1,5^ on behalf of the CKB-Air Collaborative Group

^1^Clinical Trial Service Unit and Epidemiological Studies Unit, Nuffield Department of Population Health, University of Oxford, UK

^2^Jockey Club School of Public Health and Primary Care, The Chinese University of Hong Kong, Hong Kong SAR

^3^Chinese Academy of Medical Sciences, China

^4^Faculty Research Centre for Intelligent Healthcare, Faculty of Health and Life Sciences, Coventry University, UK

^5^MRC Population Health Research Unit, Nuffield Department of Population Health, University of Oxford, UK

^6^School of Public Health, Fudan University, China

^7^Department of Epidemiology and Biostatistics, Peking University, China

*Corresponding author

**Table of Contents**

**Appendix A: Supplementary figures**

**Figure A.1.** Ten study areas of the China Kadoorie Biobank Cohort Study

**Figure A.2.** Averaged 24-hour variation of personal PM2.5 levels across the three study sites in the warm and cool season in women

**Figure A.3.** Averaged 24-hour variation of personal PM2.5 levels across the three study sites in the warm and cool season in men

**Appendix B: Supplementary tables**

**Table B.1.** Selected baseline (2004-2008) characteristics of participants included in the warm and cool season campaign of the present pilot study (2017-2018)

**Table B.2.** Missing data in warm and cool season campaign

**Table B.3.** Mean and standard deviation of duration and proportion of recall period spent at different locations, by household visit in warm and cool season

**Table B.4.** Means and standard deviation (SD) of personal exposure to fine particulate matter (PM_2.5_, µg/m^3^) by season and study area in women and men

**Appendix C: Supplementary methods**

**Appendix D: Supplementary discussion**

**References for the appendices**

# Appendix A. Supplementary Figures

**Figure A1. Ten study areas of the China Kadoorie Biobank Cohort Study***

* Reproduced from Chen et al., 2011. Solid circles (●) are rural areas and open circles (○) are urban areas included in the China Kadoorie Biobank Study. Number of participant at baseline in each study area is shown in brackets.

**Supplementary Figure A2. Averaged 24-hour variation of personal PM_2.5_ levels across the three study sites in the warm and cool season in women***

**
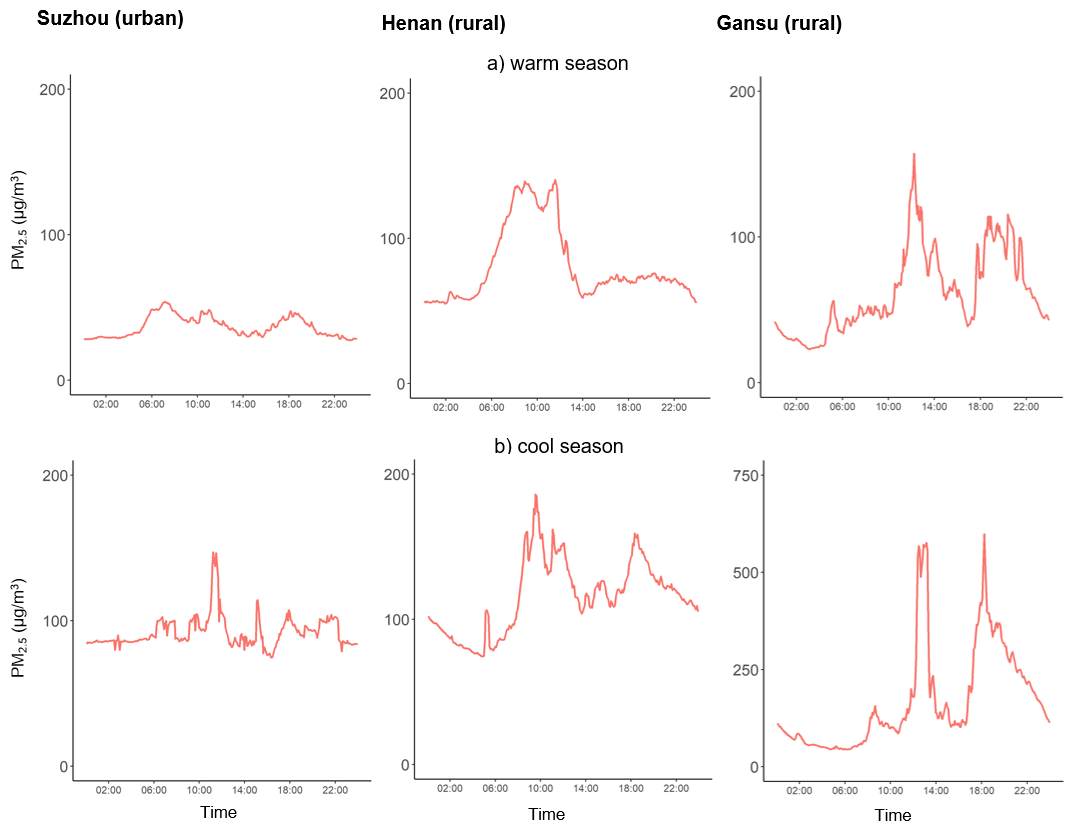
**

*Time frame displayed: from 00:00 to 24:00

**Supplementary Figure A3. Averaged 24-hour variation of personal PM_2.5_ levels across the three study sites in the warm and cool season in men***

**
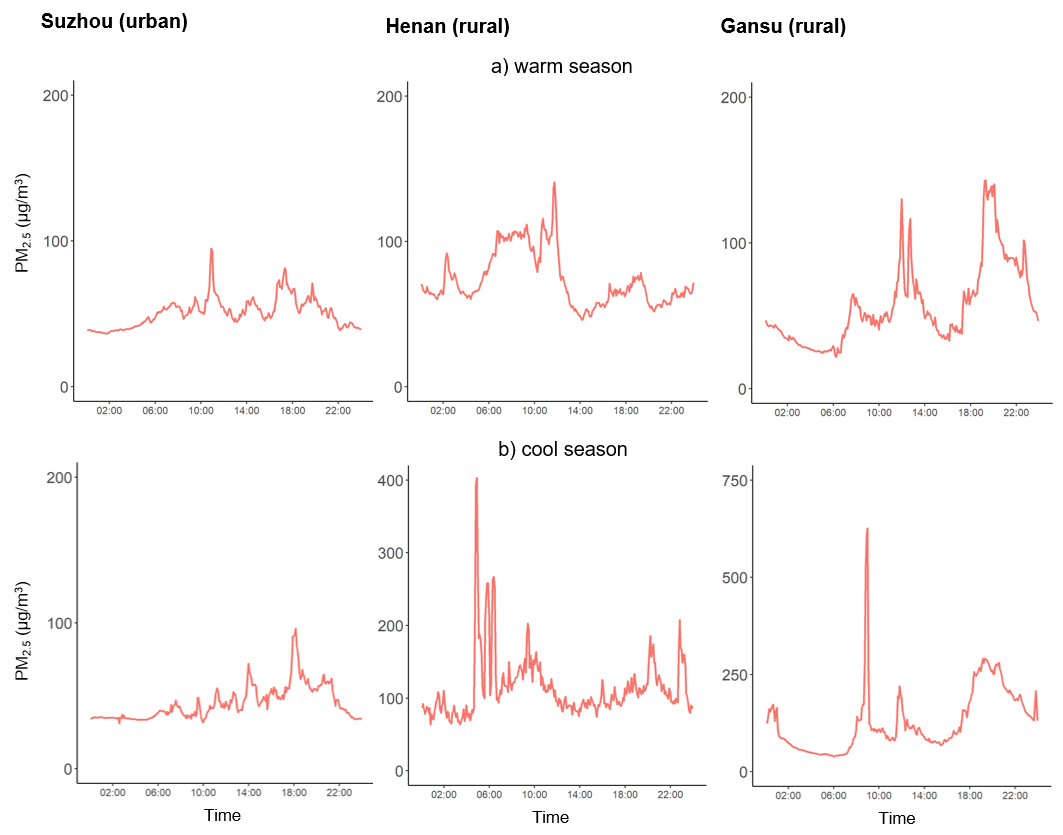
**

*Time frame displayed: from 00:00 to 24:00.

# Appendix B. Supplementary Tables

| **Table B1. Selected baseline (2004-2008) characteristics of participants included in the warm and cool season campaign of the present pilot study (2017-2018)*** | | | |
| --- | --- | --- | --- |
| **Baseline characteristics** | **Warm season (N = 451) n (%)** |  | **Cool season (N = 450) n (%)** |
| **Age - years** |  |  |  |
| < 40 | 79 (17.6) |  | 83 (18.4) |
| 40-49 | 203 (45.1) |  | 202 (44.9) |
| 50-59 | 158 (35.1) |  | 154 (34.2) |
| 60-69 | 10 (2.2) |  | 11 (2.4) |
| ≥ 70 |  |  |  |
| **Female** | 329 (73.1) |  | 326 (72.4) |
| **Education** |  |  |  |
| No formal | 111 (24.7) |  | 112 (24.9) |
| Primary school | 164 (36.4) |  | 159 (35.3) |
| Middle school | 125 (27.8) |  | 126 (28.0) |
| High school or above | 50 (11.1) |  | 53 (11.8) |
| **Household income - Yuan/ year** |  |  |  |
| < 10,000 | 208 (46.2) |  | 209 (46.4) |
| 10,000 - 19,999 | 95 (21.1) |  | 95 (21.1) |
| ≥ 20,000 | 147 (32.7) |  | 146 (32.4) |
| **Smoking** |  |  |  |
| Never-regular | 341 (75.8) |  | 338 (75.1) |
| Occasional | 12 (2.7) |  | 11 (2.4) |
| Ex-regular | 14 (3.1) |  | 13 (2.9) |
| Current-regular | 83 (18.4) |  | 88 (19.6) |
| **Alcohol consumption** |  |  |  |
| Never-regular | 232 (51.6) |  | 230 (51.1) |
| Occasional or monthly | 160 (35.6) |  | 157 (34.9) |
| Ex-regular or reduced intake | 14 (3.1) |  | 14 (3.1) |
| Current-regular | 44 (9.8) |  | 49 (10.9) |
| **Primary cooking fuel** |  |  |  |
| No cooking | 103 (22.9) |  | 108 (24.0) |
| Clean fuels | 111 (24.7) |  | 112 (24.9) |
| Coal | 167 (37.1) |  | 162 (36.0) |
| Wood | 64 (14.2) |  | 63 (14.0) |
| Other | 5 (1.1) |  | 5 (1.1) |
| **Primary heating fuel** |  |  |  |
| No heating | 128 (28.4) |  | 128 (28.4) |
| Clean fuels | 33 (7.33) |  | 31 (6.9) |
| Coal | 212 (47.1) |  | 214 (47.6) |
| Wood | 67 (14.9) |  | 67 (14.9) |
| Other | 10 (2.2) |  | 10 (2.2) |
| **Self-reported poor health** | 54 (12.0) |  | 51 (11.3) |
| **Prevalent diabetes^†^** | 29 (6.4) |  | 30 (6.7) |
| **Prevalent cardiovascular disease**^‡^ | 8 (1.8) |  | 6 (1.3) |
| **Prevalent hypertension** | 47 (10.4) |  | 46 (10.2) |

* All χ^2^ p-value comparing between the two samples were >0.05.
^†^ Self-reported prior doctor diagnosis of diabetes or screen-detected diabetes based on random blood glucose test.
^‡^ Self-reported prior doctor diagnosis of coronary heart disease, transient ischaemic attack or stroke.

| **Table B2. Missing data in warm and cool season campaign** | | | | | | | | | |
| --- | --- | --- | --- | --- | --- | --- | --- | --- | --- |
|  | **Warm season  (n = 451)** | | | |  | **Cool season  (n = 450)** | | | |
|  | **Thu** | **Fri** | **Mon** | **Fri** |  | **Thu** | **Fri** | **Mon** | **Fri** |
| **Types of missing** |  |  |  |  |  |  |  |  |  |
| **Did not undertake time-activity questionnaire** | 0 | 1 | 3 | 0 |  | 0 | 1 | 2 | 0 |
| **Did not undertake household questionnaire** | - | - | - | - |  | 1 | - | - | - |
| **Only participated in one season** | 37 | | | |  | 37 | | | |

| **Table B3. Mean and standard deviation of duration and proportion of recall period spent at different locations, by household visit in warm and cool season** | | | | | | |
| --- | --- | --- | --- | --- | --- | --- |
|  | **Weekday** | | **Weekend** | | **Overall** | |
|  | **n*** | **Median (IQR)** | **n*** | **Median (IQR)** | **n*** | **Median (IQR)** |
| **WARM SEASON** |  |  |  |  |  |  |
| **Time spent in kitchen - hours** | 328 | 1.7 (1.0-2.3) | 327 | 2.0 (1.0-2.7) | 655 | 1.8 (1.0-2.5) |
| **% of recall period spent in kitchen** |  | 6.8 (4.2-9.7) |  | 7.8 (4.1-10.9) |  | 7.3 (4.2-10.2) |
| **Time spent at home - hours** | 449 | 20.3 (16.2-23.3) | 445 | 20.2 (16.5-23.6) | 894 | 20.3 (16.5-23.5) |
| **% of recall period spent at home** |  | 83.4 (66.7-95.8) |  | 83.4 (68.8-95.9) |  | 83.4 (67.3-95.9) |
| **Time spent indoor - hours** | 418 | 21.5 (18.0-23.8) | 421 | 21.8 (19.0-24.0) | 839 | 21.7 (18.5-24.0) |
| **% of recall period spent indoor** |  | 89.6 (74.5-97.9) |  | 89.7 (77.1-98.7) |  | 89.6 (75.5-98.0) |
| **Time spent outdoor - hours** | 333 | 4.0 (2.0-7.0) | 318 | 4.0 (2.0-6.5) | 651 | 4.0 (2.0-6.8) |
| **% of recall period spent outdoor** |  | 15.8 (8.00-29.2) |  | 16.5 (8.16-26.2) |  | 16.4 (8.05-28.1) |
| **Time spent at roadside - hours** | 191 | 2.0 (1.0-4.0) | 173 | 2.0 (1.0-4.0) | 364 | 2.0 (1.0-4.0) |
| **% of recall period spent at roadside** |  | 8.3 (4.2-16.2) |  | 8.3 (4.1-16.5) |  | 8.3 (4.2-16.4) |
| **Time spent for road surface transportation - hours** | 112 | 0.7 (0.5-1.3) | 96 | 0.9 (0.5-1.3) | 208 | 0.7 (0.5-1.3) |
| **% of recall period spent for road surface transportation** |  | 2.8 (2.0-5.5) |  | 3.6 (2.1-5.5) |  | 2.9 (2.0-5.5) |
| **COOL SEASON** |  |  |  |  |  |  |
| **Time spent in kitchen - hours** | 363 | 2.5 (2.0-3.0) | 365 | 2.5 (1.8-3.0) | 728 | 2.5 (2.0-3.0) |
| **% of recall period spent in kitchen** |  | 10.2 (7.8-12.5) |  | 10.3 (7.5-12.5) |  | 10.3 (7.6-12.5) |
| **Time spent at home - hours** | 449 | 21.5 (18.3-24.0) | 446 | 21.0 (18.3-24.0) | 895 | 21.3 (18.3-24.0) |
| **% of recall period spent at home** |  | 88.0 (75.0-100) |  | 87.5 (75.0-100) |  | 87.5 (75.0-100) |
| **Time spent indoor - hours** | 389 | 23.0 (20.5-24.0) | 386 | 23.0 (20.0-24.0) | 775 | 23.0 (20.0-24.0) |
| **% of recall period spent indoor** |  | 95.8 (83.6-100) |  | 93.8 (82.5-100) |  | 94.7 (83.3-100) |
| **Time spent outdoor - hours** | 267 | 3.0 (1.5-5.0) | 269 | 3.0 (1.5-5.5) | 536 | 3.0 (1.5-5.4) |
| **% of recall period spent outdoor** |  | 12.5 (6.12-20.8) |  | 12.5 (6.25-22.8) |  | 12.5 (6.25-22.2) |
| **Time spent at roadside - hours** | 144 | 2.0 (1.0-3.7) | 113 | 1.8 (1.0-3.0) | 257 | 2.0 (1.0-3.3) |
| **% of recall period spent at roadside** |  | 8.2 (4.1-15.2) |  | 7.2 (4.1-12.5) |  | 7.8 (4.1-13.4) |
| **Time spent for road surface transportation - hours** | 75 | 1.0 (0.5-1.3) | 76 | 1.0 (0.5-1.3) | 151 | 1.0 (0.5-1.3) |
| **% of recall period spent for road surface transportation** |  | 4.0 (2.1-5.5) |  | 4.0 (2.1-5.5) |  | 4.0 (2.1-5.5) |

*n: number of participants with non-zero duration reported for the corresponding location.

| **Table B4. Means and standard deviation (SD) of personal exposure to fine particulate matter (PM_2.5_, µg/m^3^) by season and study area in women and men** | | | | | | |
| --- | --- | --- | --- | --- | --- | --- |
|  | **WOMEN** | |  | **MEN** | |  |
|  | **Mean** | **SD** |  | **Mean** | **SD** |  |
| **Warm season** |  |  |  |  |  |  |
| **Suzhou (urban)** | 36.6 | 61.1 |  | 50.9 | 117.2 |  |
| **Gansu (rural)** | 61.7 | 296.6 |  | 57.0 | 182.9 |  |
| **Henan (rural)** | 80.3 | 155.8 |  | 73.3 | 109.0 |  |
| **Cool season** |  |  |  |  |  |  |
| **Suzhou (urban)** | 91.9 | 342.8 |  | 45.1 | 89.4 |  |
| **Gansu (rural)** | 171.3 | 1716.9 |  | 129.7 | 664.2 |  |
| **Henan (rural)** | 114.9 | 195.2 |  | 114.4 | 585.4 |  |

# Appendix C: Supplementary methods

*a) Conventional gravimetric sampling for ambient air pollution*

Gravimetric sampling for the calibration of ambient monitors was done following established procedures described previously (Tong et al., 2018; Cao et al., 2005). Briefly, a mini-vol portable sampler with PM_2.5_ impactor (Airmetrics, Eugeene, OR, USA) was co-located with the ambient monitors to measure 24 hour average mass concentration, with PM_2.5_ samples collected on 47 mm quartz micro-fibre filters (Whatman, Maidstone, Kent, UK). A microbalance (Sartorius AG, Model ME 5-0CE, Goettingen, Germany) with 1 µg precision was used for filter weighting. The balance was calibrated with 200 and 100 Class 1 standard weights and tare was set before weighing each batch of filters. Triplicate filter weights were determined in a temperature (20-25 °C) and relativity humidity (35 ± 5%) controlled environment. Average of the triplicate post- and pre-weights were used to calculate mass concentrations

*b) Measurement of biomarkers*

In the same visit where time-activity questionnaire interviews were conducted, exhaled carbon monoxide (COex), blood oxygen saturation (SpO_2_) and heart rate were measured twice for each participant, using MicroCO meter (CareFusion, Basingstoke, UK) and Onyx Vantage 9590 pulse oximeter (Nonin Medical Inc., Plymouth, MN, USA), respectively.

Details of the procedures to take COex measurements have been published elsewhere (Zhang et al., 2013). Briefly, participants were instructed to fully inhale and hold their breath for 20 seconds (or, among elderly, at least 15 seconds if longer time was not achievable) then gradually and fully exhale out to the mouthpiece of the MicroCO meter while making sure there was no sideways air leakage out of the mouthpiece. Participants were allowed to take several practice blows until they were familiar with the technique, but each actual blow had to be made only after at least three minutes of resting (to allow the carboxyhaemoglobin level in the alveoli to reach equilibrium). All MicroCO meters were calibrated using standard 20 ppm CO gas at the start of the fieldwork in each study site in each season.

For SpO_2_ and heart rate, the participants should have been in resting position for at least three minutes and they were asked to insert their index finger flatly into the oximeter and wait until a green indicator light flashes for four consecutive seconds. The details of biomarker measurements will also be covered in a separate analytical study investigating the associations between different participant characteristics (e.g. smoking, cooking fuel use, ventilation) and measured air pollution and biomarkers levels.

**Appendix D: Supplementary discussion**

**Supplementary discussion [1] – future investigation on the roles of personal characteristics on PM_2.5_ exposure and the relationships between personal, household and ambient levels of PM_2.5_**

Mixed-effects regression will be used to investigate the roles of personal characteristics (e.g. sex, socioeconomic status, lifestyle, fuel use, time-activity patterns) on personal PM_2.5_ exposure, with least-square mean PM_2.5_ levels estimated for each categories of the above characteristics. Of particular interest is how the seasonal changes in fuel use, time-activity, and ambient temperature influence the relationships of personal characteristics with personal PM_2.5_ exposure.

With the PM_2.5_ data measured using the same type of light-scattering sensor across personal, household and ambient environments, the relationship between PM_2.5_ levels recorded across different environments will be examined using mixed-effects regressions. The correlation between the PM_2.5_ levels (both in overall 24-hour average and time-series format) recorded across different environments will be assessed, and inter-environment exposure ratio will be derived (e.g. personal : kitchen, household : ambient). These would improve our understanding on the complex dynamics of air pollution exposure, particularly the roles of ambient air pollution sources in household air pollution, and vice versa.

**Supplementary discussion [2] - PM_2.5_ exposure modelling strategies for future analyses**

Ultimately, we aim to develop prediction models of personal air pollution exposure in an adequately large subset (~2000-3000 households) of the CKB and apply such models to the 0.5 million participants for epidemiological analysis. To demonstrate the feasibility and value of such method, predictive models of personal exposure to PM_2.5_ will be developed by integrating pilot study data on measured exposure, self-reported household and time-activity questionnaire, and measured biomarkers, wherever appropriate, using a mixed-effect model framework as adopted by Chen et al. (Chen et al., 2018). There will be three key sets of models with increasing number of predictors. The basic models include the key variables (e.g. sex, primary cooking fuel, ventilation) commonly available in the World Health Organization HAP database used for global HAP exposure estimation (Shupler et al., 2018); the secondary models will involve a larger list of relevant covariates that had been recorded in the whole CKB population at baseline; and the tertiary models will include additional factors collected in CKB-Air for further enhancements. The factors to be included in the secondary and tertiary models will be added using a stepwise approach to understand their role in predicting air pollution exposure. The models can be compared for their predictive power, and the relative improvements attributed to the inclusion of various factors will elucidate the limitation of the current global HAP exposure estimates and inform the design of future questionnaires.

Although the models to be developed from this pilot study may not be immediately generalisable to the whole CKB population (or beyond China), they will demonstrate the feasibility and value of moving beyond self-reported HAP-exposure or conventional AAP modelling. Furthermore, the modelling pipelines established will facilitate a rapid up-scaling of this study to a larger sample size or across different populations in the future.

**References for the appendices**

Tong X, Wang B, Dai W-T, Cao J-J, Ho SSH, Kwok TCY, et al., 2018. Indoor air pollutant exposure and determinant factors controlling household air quality for elderly people in Hong Kong. Air Qual Atmos Health. 11 (6), 695-704.

Cao JJ, Lee SC, Chow JC, Cheng Y, Ho KF, Fung K, et al, 2005. Indoor/outdoor relationships for PM2.5 and associated carbonaceous pollutants at residential homes in Hong Kong - case study. Indoor Air. 15 (3), 197-204.

Zhang Q, Li L, Smith M, Guo Y, Whitlock G, Bian Z, et al., 2013. Exhaled carbon monoxide and its associations with smoking, indoor household air pollution and chronic respiratory diseases among 512 000 chinese adults. Int J Epidemiol. 42 (5), 1464-1475.

Chen C, Cai J, Wang C, Shi J, Chen R, Yang C, et al., 2018. Estimation of personal PM2.5 and BC exposure by a modeling approach - Results of a panel study in Shanghai, China. Environ Int. 118, 194-202.

Shupler M, Godwin W, Frostad J, Gustafson P, Arku RE, Brauer M, 2018. Global estimation of exposure to fine particulate matter (PM2.5) from household air pollution. Environ Int. 120, 354-363.

Chen Z, Chen J, Collins R, Guo Y, Peto R, Wu F, et al., 2011. China Kadoorie Biobank of 0.5 million people: survey methods, baseline characteristics and long-term follow-up. Int J Epidemiol. 40 (6), 1652-1666.
